# Non-Coding RNAs Derived from Extracellular Vesicles Promote Pre-Metastatic Niche Formation and Tumor Distant Metastasis

**DOI:** 10.3390/cancers15072158

**Published:** 2023-04-05

**Authors:** Jin Cheng, Kun Zhang, Chunhui Qu, Jinwu Peng, Lifang Yang

**Affiliations:** 1 Department of Oncology, Key Laboratory of Carcinogenesis and Cancer Invasion of Ministry of Education, National Clinical Research Center for Geriatric Disorders, Xiangya Hospital, Central South University, Changsha 410078, China; 2 Cancer Research Institute, School of Basic Medicine Science, Central South University, Changsha 410078, China; 3 Department of Pathology, Xiangya Hospital, Central South University, Changsha 410078, China; 4 Department of Pathology, Xiangya Changde Hospital, Changde 415000, China

**Keywords:** extracellular vesicles, non-coding RNAs, pre-metastatic niche, distant metastasis

## Abstract

**Simple Summary:**

Tumor cells generate extracellular vesicles, which exert a critical role in intercellular communication through the delivery of their cargo, thus regulating many cell functions to promote the tumor’s progression. This review focuses on the formation of pre-metastatic niche, which is a key process in the early metastasis of tumor, and provides an overview of the role and mechanism of non-coding RNA derived from extracellular vesicles in mediating tumor-distant metastasis, and its predictive value for tumor diagnosis and evaluation as a potential target for anti-tumor metastasis.

**Abstract:**

Metastasis is a critical stage of tumor progression, a crucial challenge of clinical therapy, and a major cause of tumor patient death. Numerous studies have confirmed that distant tumor metastasis is dependent on the formation of pre-metastatic niche (PMN). Recent studies have shown that extracellular vesicles (EVs) play an important role in PMN formation. The non-coding RNAs (ncRNAs) derived from EVs mediate PMN formation and tumor-distant metastasis by promoting an inflammatory environment, inhibiting anti-tumor immune response, inducing angiogenesis and permeability, and by microenvironmental reprogramming. Given the stability and high abundance of ncRNAs carried by EVs in body fluids, they have great potential for application in tumor diagnosis as well as targeted interventions. This review focuses on the mechanism of ncRNAs derived from EVs promoting tumor PMN formation and distant metastasis to provide a theoretical reference for strategies to control tumor metastasis.

## 1. Introduction

Tumor-distant metastasis is a complicated multi-stage process [1]. The metastatic tumor cells detach from the primary site, break through the basement membrane and enter the circulation, and rapidly proliferate in the secondary site to form metastases under the appropriate microenvironment [2]. In 1889, Paget’s “seed and soil” hypothesis suggested that a suitable microenvironment for tumor cell growth is a prerequisite for metastasis of disseminated tumor cells to distant organs [3]. In 2005, Kaplan et al. further proposed the concept of pre-metastatic niche, suggesting that before metastasis occurs, tumors induce adaptive changes in the microenvironment of distant organs, making it favorable for tumor cells to colonize and survive and then form metastatic foci. This preformed supportive microenvironment is called “pre-metastatic niche” (PMN) [4,5,6]. Five characteristics of PMN have been identified, including inflammation, immunosuppression, angiogenesis and vascular permeability, organ tropism, and reprogramming [7,8].

Extracellular vesicles (EVs) are lipid bilayer vesicles released from cells, and their bilayer membranes prevent the contents from being degraded by exogenous nucleases and proteases, facilitating the long time and long distance transport of biomolecules [9,10]. EVs lack a functional nucleus and are incapable of self-replication, and are vesicles that are continuously released by cells and that participate in various physiological and pathological processes through the delivery of their cargo [11,12,13,14]. EVs are present in many body fluids, such as blood, urine, and saliva, and can be recognized by their unique biological and secretory mechanisms [15]. In recent years, a lot of research shows that EVs promote PMN formation and distant metastasis by transporting functional molecules, including nucleic acids (e.g., DNA, mRNA and non-coding RNAs (ncRNAs)), proteins (e.g., cytokines, membrane receptors and receptor ligands), lipids, and so on [16,17].

**Figure 1:**
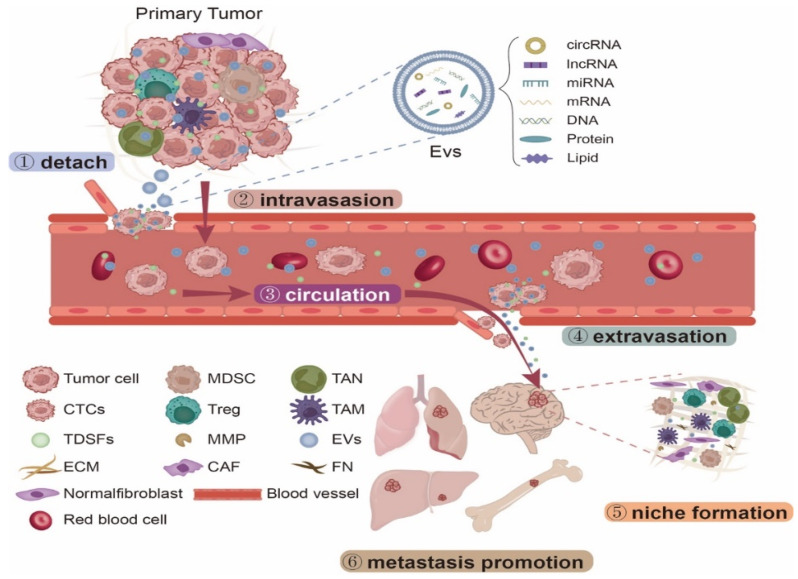
NcRNAs derived from EVs are involved in multi-stage process of tumor-distant metastasis, including the tumor cells detaching from the primary site, breaking through the basement membrane and entering the circulation, and inducing the formation of PMN, thereby promoting tumor metastases to form in the target organ.

Notably, ncRNAs play a significant role in regulating tumor PMN formation and distant metastasis [18]. NcRNA components include microRNA (miRNA), long non-coding RNA (lncRNA) and circular RNA (circRNA) [19,20]. In addition to participating in the regulation of intracellular gene expression [21,22,23], these ncRNAs can also be selectively packaged into EVs and transported to distant tissue cells, where they participate in distant metastasis by mediating a range of gene expression and signaling pathways [24]. NcRNAs derived from EVs are involved in several stages of tumor metastasis [25,26]. Firstly, the tumor cells detach from the primary site and subsequently destroy the intercellular matrix and stimulate the formation of capillaries [27]. Afterwards, the primary tumor cells break through the basement membrane and enter the capillaries and follow the blood circulation to all parts of the body [28,29]. The surviving tumor cells stay in the right conditions, cross the capillary walls, and leave the blood circulation [30]. In the perivascular setting, ncRNAs derived from EVs interact with various factors in the microenvironment to promote the formation of PMN that facilitate tumor colonization. Eventually, metastases form in the target organ [31,32]. In conclusion, ncRNAs derived from EVs play a relevant function as metastasis promoters during tumor-distant metastasis [33,34] (Figure 1).

## 2. NcRNAs Derived from EVs Promote PMN Formation

There is growing evidence that ncRNAs carried by EVs can promote tumor-distant metastasis by establishing PMN at distal metastatic sites in multiple ways, including promoting upregulation of inflammatory molecules; induction of immunosuppression; impairment of T cells; NK cell and antigen presentation; increase in angiogenesis and vascular permeability; activation of metabolic reprogramming; and remodeling of stromal cells and the extracellular matrix (ECM) [35,36,37,38].

### 2.1. NcRNAs Derived from EVs Upregulate the Inflammatory Molecules

Chronic inflammation is a driver that triggers tumor progression and metastasis. Inflammatory cells and inflammatory molecules are principal components of inflammatory microenvironment [39,40]. Additionally, ncRNAs derived from EVs play an important role in contributing to the inflammatory microenvironment at the site of tumor-distant metastasis. For example, non-small-cell lung cancer (NSCLC) cells released exosomal miR-21 and miR-29a, which activated NF-κ B by binding to TLR7 and TLR8 in macrophages, and eventually led to increased secretion of inflammatory cytokines (such as IL-6 and TNF-α), thus promoting the formation of inflammatory environment at tumor metastasis sites [41]. The combination of exosomal miR-21 and TLR7 also mediated the polarization of hepatic macrophages and formed the pro-inflammatory phenotype of IL-6 secretion, which provided a favorable environment for liver metastasis of colorectal cancer (CRC) [42]. Alveolar epithelial type II cells took up the breast-cancer-derived exosomal miR-200b-3p, and induced high expression of chemokine ligand 2 (CCL2), S100A8/9, matrix metalloproteinase 9 (MMP9), and colony-stimulating factor 1 (CSF-1) to recruit myeloid-derived suppressor cells (MDSCs) and promote inflammatory PMN formation [43]. These studies fully illustrate that the ncRNA carried by EVs contributes to the upregulation of pro-inflammatory cytokines, promotes the formation of inflammatory microenvironment at the site of distant tumor metastasis, and plays a significant role in the initiation of PMN.

### 2.2. NcRNA Carried by EVs Induce Immunosuppression

NcRNA carried by EVs can create an immunosuppressive environment for tumor cell growth in vivo by inducing immunosuppressive cell (e.g., MDSCs) populations and suppressing anti-tumor immune responses of immune cells (e.g., dendritic cell DCs, natural killer NK cells, T lymphocytes), thus enabling tumor immune escape and promoting distant tumor cell metastasis [44,45]. For instance, exosomal miR-203 secreted by CRC cells was absorbed by monocytes and promoted their differentiation into M2-type tumor-associated macrophages (TAM), which induced the immunosuppressive microenvironment formation at pre-metastatic sites, thus promoting the liver metastasis of CRC [46]. Another study also found that colon-cancer-cell-derived exosomal miR-934 induced macrophage M2 polarization through down regulation of PTEN expression and activation of PI3K/AKT signaling pathways, and M2-type macrophages secreted CXCL13 to affect the liver immune status, induce PMN formation, and promote liver metastasis of colon cancer [47]. EVs derived from hypoxia pancreatic adenocarcinoma cells transported miR-301a-3p in a HIF1a- or HIF2a-dependent manner, induced macrophage M2 polarization through activation of the PTEN/PI3Kγ signaling pathway, and thus promoted lung metastasis of pancreatic adenocarcinoma cells [48]. In addition, pancreatic-adenocarcinoma-cell-derived exosomal miR-212-3p could inhibit antigen presentation from dendritic cells to T lymphocytes by suppressing the expression of MHC II-specific regulatory factor X-associated protein (RFXAP), thereby inducing immune tolerance of PMN and promoting tumor metastasis [49]. Lin28B reduced the expression of let-7s in breast cancer exosomes and regulated the expression of CXCL, IL-6, and IL-10 in neutrophils, resulting in immunosuppressive PMN, thus promoting lung metastasis of breast cancer [50]. These findings suggested that ncRNA carried by EVs can inhibit the function of the immune system in many ways, which are beneficial to the immune escape of tumor cells and play a crucial role in the formation of PMN.

### 2.3. NcRNA Carried by EVs Promote Angiogenesis and Vascular Permeability

Angiogenesis is the process of re-formation of blood vessels from endothelial progenitor cells or germination of new vessels from blood vessels [51]. The generation of neovascularization can meet the nutrition required for the rapid growth of tumor cells after colonization. At the same time, the microenvironment that promotes angiogenesis also enhances vascular permeability, thus facilitating the extravasation of tumor cells and allowing them to enter the pre-metastatic organs via the blood circulation [52]. For example, miR-939 was highly expressed in breast cancer cells and transferred extracellularly via exosomes, leading to downregulation of VE-calmodulin in vascular endothelial cells and increasing vascular permeability, thus promoting breast cancer metastasis [53]. Exosomal miR-181c from breast cancer cells targeted PDPK1 in brain endothelial cells and regulated the localization of tight junction proteins N-calmodulin and actin, thereby increasing vascular permeability and destabilizing the blood-brain barrier (BBB), leading to brain metastasis [54]. Neutral sphingomyelinase 2 (nSMase2) could promote breast cancer cells to secrete exosomes loaded with miR-210, to enhance the angiogenic activity of endothelial cells, thereby increasing lung metastasis of breast cancer [55]. EV-derived miR-23a was highly enriched in nasopharyngeal carcinoma (NPC) tissues during metastatic or pre-metastatic stages, and its levels in NPC correlated with microvessel density. Further studies revealed that miR-23a could promote NPC metastasis by directly targeting testis-specific gene antigen (TSGA10) in endothelial cells to regulate angiogenesis [56]. In addition, bone marrow-derived suppressor cell (MDSC) exosomal miR-126a promoted tumor angiogenesis in an S100A8/A9-dependent manner, thereby facilitating lung metastasis of breast tumors [57]. It was confirmed that ncRNAs carried by EVs also play an essential regulatory role in angiogenesis and vascular permeability in PMN formation. For instance, CRC-derived exosomal miR-25-3p regulated the expression of VEGFR2, ZO-1, Occludin and Claudin5 in endothelial cells through KLF2/4, and promoted angiogenesis and disrupted the tight junctions of vascular endothelial cells, and further induced vascular permeability in mouse liver and lung, and thus promoted the formation of PMN at these sites [58]. The miR-105 carried by EVs secreted from breast cancer cells disrupted the barrier function of the endothelial cell monolayer by downregulating the tight junction protein ZO-1, thereby increasing vascular permeability in PMN, leading to promotion of tumor metastasis [59]. The exosomal miR-638 derived from hepatocellular carcinoma (HCC) cell HuH-7M inhibited VE-calmodulin and ZO-1 expression, and further diminished the endothelial junctional integrity, thereby enhancing vascular permeability and initiating PMN, thus promoting tumor metastasis [60]. Studies have found that the exosomal miR-3157-3p, derived from NSCLC cells, was involved in the formation of PMN and lung metastasis. The mechanism was that miR-3157-3p could regulate the expression of VEGF/MMP2/MMP9 in endothelial cells through TIMP/KLF2, thereby promoting angiogenesis and increasing vascular permeability [61]. The above studies fully demonstrated that ncRNAs carried by EVs released from tumor cells and cells of other origins could participate in angiogenesis and increase vascular permeability in PMN formation, create a microenvironment rich in blood supply in distant metastatic organs, thus facilitating the colonization and survival of tumor cells.

### 2.4. NcRNA Carried by EVs Promote Reprogramming

Reprogramming refers to altering the biological behavior of cells through epigenetic modifications without altering the gene sequence [62]. Studies have shown that reprogramming is closely related to tumorigenesis and progression, and also plays an important role in PMN formation and distant metastasis [63,64]. Metabolic reprogramming and matrix reprogramming are two important aspects of ncRNAs derived from EVs involved in PMN formation.

#### 2.4.1. Metabolic Reprogramming

NcRNA carried by EVs can reprogram glucose metabolism at pre-metastatic sites to promote distant metastasis by increasing nutrient uptake of tumor cells. Fong et al. found that breast cancer cells could secrete exosomes carrying high levels of miR-122, which were taken in by fibroblasts in the PMN of the brain and lung. The uptake of miR-122 by fibroblasts downregulated the expression of pyruvate kinase, which inhibited glucose uptake by non-tumor cells in PMN to accommodate the high energy demand of tumor cells during metastatic cell growth, that is, to promote metastasis by increasing the utilization of nutrients by tumor cells [65]. In addition, under the action of p-STAT3/miR-193a-3p/LAMC1 axis, preadipocytes enriched in the peritoneum differentiate into mature adipocytes for metabolic reprogramming, thus promoting PMN formation in the peritoneal microenvironment and colonization and metastasis of gastric cancer (GC) cells to the peritoneum [66]. Melanoma-cell-derived exosomal miR-155 and miR-210 targeted adrenal fibroblasts (HADF), leading to an increase in their glycolysis and a decrease in oxidative phosphorylation (OXPHOS), resulting in the formation of PMN that contributes to melanoma metastasis [67]. The above studies suggest that ncRNAs carried by EVs could be involved in the metabolic reprogramming of PMN, transforming its metabolic environment into a microenvironment favorable to tumor cell proliferation, thereby promoting the occurrence of distant metastasis.

#### 2.4.2. Matrix Reprogramming

**Figure 2:**
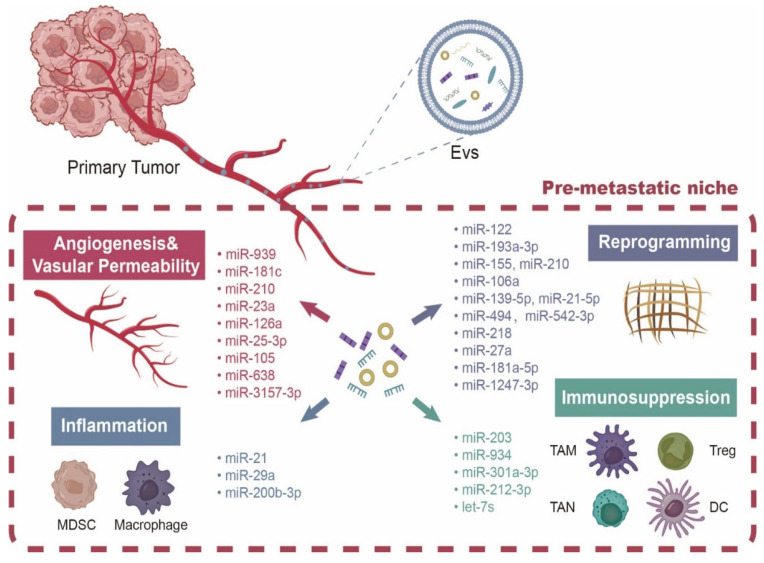
NcRNAs derived from EVs can establish PMN in distal part in a number of ways, including promoting inflammatory molecule increase; induced immunosuppressive or immune surveillance, leading to T cell, DC cell, and antigen-presenting impairment; angiogenesis and vascular permeability increase; and by activating metabolic reprogramming and stromal cell and extracellular matrix (ECM) reprogramming.

ECM and stromal cells are the most important parts of tumor microenvironment (TME), which provide support and nutrition for the growth and metastasis of tumor cells [68]. Studies have shown that ncRNAs carried by EVs can mediate the deposition of fibronectin (FN), collagen, MMPs, and lysyl oxidase (LOX) in the microenvironment of pre-metastatic organ to remodel the ECM and promote tumor cell adhesion and colonization. For example, the GC-cell-derived exosomal miR-106a increased fibronectin deposition, and remodeled the ECM to establish proper PMN and promote GC peritoneal metastasis by targeting Smad7 and TIMP2 in peritoneal mesothelial cells to activate the TGF-β pathway [69]. Prostate-cancer-cell-derived exosomal miR-139-5p and miR-21-5p remodeled ECM by promoting MMP-2 and MMP-13 expression in stromal cells, thereby contributing to PMN formation and distant metastasis of prostate cancer [70]. EV-loaded miR-494 and miR-542-3p in rat pancreatic cancer cells promoted lung metastasis by targeting calmodulin-17 and upregulating MMP 2 and 9 expression in pre-metastatic lung stromal cells to remodel the stromal microenvironment in PMN [71]. In addition, miR-218 carried by EVs derived from metastatic breast cancer targeted COL1A1, YY1, and INHBB, and then inhibited collagen expression in osteoblasts, resulting in an osteolytic microenvironment conducive to metastatic colonization and promoting PMN formation and bone metastasis [72]. EV-loaded ncRNA may also promote ECM remodeling and PMN formation by directly targeting other non-immune stromal cells, such as normal fibroblasts (NFs) [73]. GC-cell-derived exosomal miR-27a induced NF transformation into tumor-associated fibroblasts (CAFs) by downregulating CSRP2 expression, thereby promoting lung metastasis of GC [74]. The miR-181a-5p carried by EVs derived from highly metastatic CRC cells could activate hepatic stellate cells (HSCs) by targeting SOCS3 and activating the IL6/STAT3 signaling, leading to TME reprogramming and PMN formation, thereby promoting liver metastasis of CRC [75]. Exosomal miR-1247-3p from highly metastatic HCC cell directly targeted B4GALT3 in PMN, leading to activation of NFs as CAFs by β1-integrin-NF-κB signaling pathway in NFs. CAFs further promoted lung metastasis of HCC through secreting IL-6 and IL-8 [76]. The above studies suggest that ncRNAs carried by EVs can remodel the stromal microenvironment at distant metastatic sites or induce changes in normal stromal cells that favor PMN formation, and support tumor cell colonization and metastasis. Above all, ncRNAs derived from EVs promote formation of PMN (Figure 2).

## 3. NcRNAs Derived from EVs Have Organotropism

Prior to the onset of distant metastasis, primary tumor cells secrete EVs into the circulation as messengers. These EVs educate recipient cells in specific target organs with bioactive cargos, followed by organotropic colonization and proliferation of tumor cells [77,78,79]. More and more studies have shown that ncRNA-rich EVs can drive metastasis in lung, liver, bone and brain, which are very common sites for organ-specific metastasis [80,81]. Next, we will discuss the organotropism of ncRNAs derived from EVs in tumor-distant metastasis.

### 3.1. Lung Metastasis

The lung is a more common site of metastasis for many different tumors, including breast cancer and gastrointestinal tumors [82]. In patients with gastric cancer, their serum exosomal circFCHO2 was highly expressed. CircFCHO2 activated the JAK1/STAT3 pathway by sponging miR-194-5p, thereby promoting cell growth and lung metastasis in GC [83]. EVs containing lncRNA SNHG16 were highly expressed in breast cancer cells and tissues. SNHG16 promotes lung metastasis in breast cancer via the lncRNA SNHG16/miR-892b/PPAPDC1A axis [84]. In patients with metastatic colorectal cancer, a significant increase in the expression of exosomal miR-106b-3p has been observed. By targeting hepatoma 1 (DLC-1), exosomal miR-106b-3p induced EMT in colorectal cancer, thereby promoting lung metastasis of colorectal cancer cells [85].

### 3.2. Liver Metastasis

Liver metastases occur frequently in a variety of malignancies, including colorectal, lung, and pancreatic cancers [86,87]. Upward regulation of circ-IRAS expression in plasma exosomes from pancreatic cancer fabrics and patients with hepatic metastatic disease. Circ-IRAS entered HUVECs via pancreatic-cancer-cell-derived exosomes and significantly downregulated miR-122 and ZO-1 levels and upregulated RhoA and RhoA-GTP levels, thereby increasing endothelial monolayer permeability and promoting tumor metastasis [88]. High-throughput sequencing results showed that lncRNA-ALAHM is highly enriched in serum EVs from patients with liver metastases from lung adenocarcinoma (LUAD). LUAD-cell-derived EVs overexpressed ALAHM and promoted HGF secretion from hepatocytes by binding to AUF1, thereby promoting hepatic metastasis from LUAD cells [89]. In colorectal cancer patients, the expression of plasma exosomal miR-140-3p was significantly lower than that in healthy controls. Exosomal miR-140-3p directly targeted BCL2 and BCL9, thereby inhibiting CRC progression and promoting liver metastasis [90].

### 3.3. Bone Metastasis

Bone is the third most common site of tumor metastasis after the lung and liver [91,92,93,94]. There is now increasing evidence that EV-loaded ncRNAs play an important regulatory role in tumor bone metastasis [95]. Prostate-cancer-cell-derived exosomal lncRNA NEAT1 induced osteogenic differentiation of human-bone-marrow-derived mesenchymal stem cells (hBMSC) by upregulating RUNX2 expression through competitive binding to miR-205-5p via the SFPQ/PTBP205 axis [96]. The lncRNA NORAD loaded in extracellular vesicles of prostate cancer cells interacted with miR-541-3p and led to upregulation of PKM2, thereby enhancing the development of prostate cancer bone metastases [97]. Exosomal miR-375 was highly expressed in neuroblastoma with bone metastasis, and its downregulation of YAP1 expression promoted osteogenic differentiation of bone marrow mesenchymal stromal cells and thus bone metastasis [98].

### 3.4. Brain Metastasis

**Figure 3:**
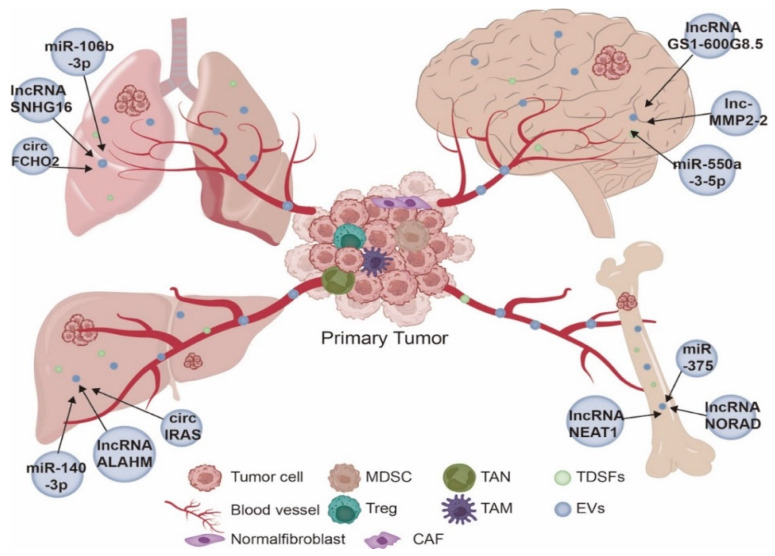
NcRNAs derived from EVs promote organ-specific metastasis. Primary tumor cells secrete EVs into the circulation as messengers. These EVs educate recipient cells in specific target organs with bioactive cargos, followed by organotropic colonization of tumor cell.

The detection of brain metastases is usually at an advanced stage of the tumor [99,100]. Brain metastases are a serious barrier to treatment for patients with solid tumors [100]. The most common primary tumor that metastasizes to the brain is lung cancer [101,102]. Non-small cell lung cancer exosomal lnc-MMP2-2 acted as a competitive endogenous RNA for miR-1207-5p to regulate the expression of EPB41L5, thereby disrupting tight junctions, increasing blood-brain barrier permeability, and thus promoting brain metastasis [103]. The lncRNA GS1-600G8.5 was highly expressed in highly brain metastatic cells and their exosomes, and it promoted brain metastasis of breast cancer cells by disrupting the blood-brain barrier [104]. MiR-550a-3-5p was significantly enriched in brain metastatic exosomes from lung cancer. The high level of exosomal MiR-550a-3-5p directly bound to YAP1 and upregulated cleaved-PARP expression, thus inducing brain metastasis in lung cancer compared to the control group [105] (Figure 3).

## 4. The Clinical Relevance of EVs Carrying ncRNA

Due to ncRNAs carried by EVs being stable, simple, easy to monitor, and tumor-specific, they are expected to be biomarkers for the assessment of PMN formation and early tumor-distant metastasis [106]. The purposeful identification and targeting of ncRNAs carried by EVs may be one of the effective methods to disrupt the formation of PMN and control tumor-distant metastasis, which has important clinical significance.

### 4.1. NcRNAs Carried by EVs May Be Biomarkers for Predicting Tumor-Distant Metastasis

The following inherent properties of ncRNAs carried by EVs give them an advantage as tumor biomarkers: (1) ncRNAs carried by EVs contain genetic components that reflect the characteristics and classes of parental cells; (2) ncRNAs carried by EVs are commonly found in human body fluids; (3) as a transport vehicle, the lipid bilayer of EVs protects its loads from degradation by nucleases and proteases [107,108,109]. Since abnormal expression of ncRNAs carried by EVs correlates with tumor phenotype, identification of abnormally ncRNAs derived from EVs in liquid biopsies can be used to monitor the metastatic potential and status of tumor patients [110,111,112]. Therefore, it is clinically important to study ncRNAs carried by EVs in different tumors that can predict tumor-distant metastasis.

#### 4.1.1. Colorectal Cancer

It was found that the exosomal miR-17-5p and miR-92a-3p were higher in the serum of CRC patients, and significantly correlated with the pathological stage and grade of CRC patients. This suggested that exosomal miR-17-5p and miR-92a-3p might provide a promising biomarker for metastatic CRC [113]. Another study showed that serum exosomal miR-19a was associated with tumor progression. In CRC patients with liver metastasis, miR-19a expression was upregulated and might be a marker for predicting liver metastasis of CRC [114]. CRC cells secreted EV; delivery miR-21 was phagocytosed by liver macrophages and established inflammatory PMN, thus playing a key role in promoting liver metastasis. In the plasma of patients with CRC liver metastasis, EVs carried higher levels of miR-21, which also could be used as a potential marker of CRC liver metastasis [42]. Exosomal miR-203 secreted by CRC cells promoted the differentiation of monocytes into M2-type TAMs, which in turn led to more liver metastases, suggesting that high miR-203 expression in serum exosomes is a novel biomarker for predicting liver metastases of CRC [46]. Exosomal miR-934 induced macrophage M2 polarization and promoted PMN formation, which led to early metastasis. Moreover, exosomal miR-934 is abnormally over-expressed in the serum of colon cancer patients with liver metastasis, and can be used as a novel biomarker to predict liver metastasis of CRC [47]. The expression level of exosomal miR-25-3p from patients with metastatic CRC is significantly higher than that in patients without metastasis, and the exosomal miR-25-3p could promote angiogenesis and increase vascular permeability and further participate in PMN formation, so the detection of miR-25-3p levels in serum exosomes will help to diagnose CRC metastasis [58]. Additionally, miR-181a-5p carried by EVs derived from highly metastatic CRC cells promoted liver metastasis of CRC by activating HSCs to establish PMN. Clinically, the levels of miR-181a-5p carried by EVs of serum was high in CRC patients and positively correlated with liver metastasis [75]. This research provides a substantial experimental basis for the clinical diagnostic application of ncRNAs carried by EVs in CRC metastasis.

#### 4.1.2. Breast Cancer

Metastasis of breast cancer is also a common problem in clinical practice [115,116]. It was found that miR-200 was enriched in serum EVs of patients with metastatic breast cancer, and it promoted mesenchymal-to-epithelial transition (MET) and led to lung metastasis by altering E-calmodulin expression, indicated that miR-200 carried by EVs might be a marker for predicting tumor metastasis [117]. Cancer-cell-derived EVs loaded with miR-105 promoted tumor metastasis by targeting ZO-1. In clinical studies, miR-105 in the serum of breast cancer patients could be used as a biomarker for the prediction or early diagnosis of breast cancer metastasis [59]. Compared with serum from breast cancer patients without bone metastases, miR-218 carried by EVs was significantly upregulated in serum from patients with bone metastases, but was not differentially expressed at other metastatic sites, showing that miR-218 is a biomarker specific for bone metastasis of breast cancer [72]. Cancer-cell-derived exosomal miR-200b-3p targeted PTEN to promote the inflammatory PMN formation at pre-metastatic sites; exosomal miR-200b-3p was highly expressed and it might be a biomarker for lung metastasis of breast cancer [43]. In addition, the abnormal expression of a large number of lncRNAs was closely related to the lung metastasis microenvironment of breast cancer. Exosomes from primary breast cancer cells loaded with lncRNAs promoted the PMN formation of the lung [118]. Low serum levels of exosomal let-7 in breast cancer promoted immunosuppressive PMN formation in the lung, and it also was a potential predictor of lung metastasis of breast cancer [50].

#### 4.1.3. Prostate Cancer

MiR-378a-3p from prostate-cancer-cell-derived EVs promoted osteolysis through activation of the Dyrk1a/Nfatc1/Angptl2 axis in bone marrow macrophages, which played an important role in prostate cancer bone metastasis and could be a potential predictor of metastatic prostate cancer [119]. MiR-425-5p was highly expressed in the exosomes secreted by metastatic prostate cancer cells and targeted the small molecule heat shock protein HSPB8, which was involved in bone metastasis. The exosomal miR-425-5p could be used as a biomarker for bone metastasis of prostate cancer [120].

#### 4.1.4. Gastric Carcinoma

In assessing the relationship between the exosomal miR-23b secreted by GC cells and clinicopathological factors in GC patients, miR-23b expression was found to correlate with tumor size, depth of infiltration, and liver metastasis and TNM stage, showing that the exosomal miR-23b could be used as a biomarker for detecting liver metastasis in GC [121]. In GC patients, the peritoneal surface is the most common site of metastasis. The frequency of peritoneal metastasis was increased in GC patients with low expression of exosomal miR-29b-3p in peritoneal fluid. Consequently, exosomal miR-29b-3p could be used as a biomarker for peritoneal metastasis of GC [122]. Among GC patients, high circ-RanGAP1 expression was closely associated with an advanced TNM stage, lymph node metastases, and worse survival. Additionally, circ-RanGAP1 silencing remarkably suppressed tumor growth and metastasis of GC in vivo. The finding suggested that circ-RanGAP1 might act as a potential prognostic biomarker [123].

#### 4.1.5. Lung Cancer

The expression of plasma exosomal miR-3157-3p in patients with metastatic NSCLC was significantly higher than that of non-metastatic patients. Exosomal miR-3157-3p participated in the formation of lung PMN before tumor metastasis by promoting angiogenesis and increasing vascular permeability, and provided a potential blood biomarker for early NSCLC metastasis [61]. Low expression levels of EV-loaded miR-192 in the serum of lung cancer patients increased angiogenesis and promoted osteolysis, further leading to bone colonization and metastasis of lung cancer. Thus, EV-loaded miR-192 has been proposed as a potential indicator of tumor metastasis [124]. LncRNA-SOX2OT was highly accumulated in exosomes derived from the peripheral blood of NSCLC patients with bone metastasis. So lncRNA-SOX2OT may serve as a treatment target in metastatic NSCLC [125].

#### 4.1.6. Hepatocellular Carcinoma

In HCC patients, serum exosomal miR-638 is enriched and has the potential to be a biomarker for early intrahepatic metastasis by disrupting the vascular barrier and thus promoting PMN formation [60]. High levels of miR-1247-3p in the serum exosomes of patients with HCC were associated with pulmonary metastasis and has potential as a predictive biomarker for tumor metastasis [76].

#### 4.1.7. Nasopharyngeal Carcinoma and Pancreatic Cancer and Cholangiocarcinoma

Serum exosomal miR-23a was highly expressed in metastatic NPC specimens compared to non-metastatic NPC or normal nasopharyngeal tissue. This suggested that exosomal miR-23a is a marker for the prediction or early diagnosis of NPC metastasis [56]. High expression of EV-loaded lncRNA-Sox2ot in plasma of patients with highly invasive pancreatic ductal adenocarcinoma (PDAC) played an important role in tumor progression and might be a potential biomarker for predicting pancreatic cancer metastasis [126]. In cholangiocarcinoma (CCA) cells, circ-CCAC1 upregulated the Yin Yang 1 (YY1) expression by sponging miR-514a-5p, thereby promoting the malignant progression of tumor cells. At the same time, circ-CCAC1 from CCA-derived EVs was transferred to endothelial cells, disrupting the integrity of the endothelial barrier and inducing angiogenesis. Circ-CCCAC1 plays a vital role in CCA carcinogenesis and metastasis, and may be an important biomarker for CCA [127]. In conclusion, this research demonstrates the possibility of using ncRNA carried by EVs as a predictive marker for tumor-distant metastasis, and further validation with large samples will greatly facilitate the clinical application of this technology (Table 1).

### 4.2. Targeted Intervention in Tumor-Distant Metastasis of ncRNAs Carried by EVs

Identification and targeting of key players in the tumor-distant metastasis microenvironment can be used to inhibit PMN formation and tumor-distant metastasis [36]. Therefore, specifically blocking the delivery of ncRNAs carried by EVs to recipient cells may be a powerful strategy to prevent tumor metastasis, and in recent years there have been many relevant and meaningful research achievements. For example, exosomal circPTGR1 derived from a high metastatic potential HCC cell promoted metastasis via the miR449a-MET pathway. Thus, circPTGR1 might be a specific target for the treatment of HCC metastasis [128]. Su et al. reported that circRNA CDR1-AS was directly transferred from HCC cells into lung cells via exosomes. Therefore, exosomal circRNA CDR1-AS may represent a future therapeutic target for HCC metastasis in clinical applications [129]. The circ-RanGAP1-mediated miR-877-3p/VEGFA axis promotes GC progression. Overexpression of miR-877-3p reversed the biological functions mediated by circ-RanGAP1 in GC cells. It suggested that circ-RanGAP1 might act as a potential therapeutic target for GC metastasis [123]. Circ-CCAC1 from CCA-derived EVs was transferred to endothelial monolayer cells, disrupting endothelial barrier integrity and inducing angiogenesis. It may be an important therapeutic target for CCA metastasis [127]. Blocking miR-25-3p carried by exosomes secreted by CRC cells reduced hepatic and pulmonary vascular permeability and subsequent PMN formation and metastasis, suggesting that miR-25-3p could be used as a therapeutic target to intervene in CRC metastasis [58]. Breast-cancer-cell-derived exosomes promoted osteoclastogenesis by transferring miR-21 to osteoclasts, thereby regulating PDCD4 protein levels and leading to PMN formation, which in turn promoted breast cancer bone metastasis. Blocking exosomal miR-21 could be a potential clinical treatment to inhibit breast cancer bone metastasis [130]. Prostate-cancer-cell-derived EVs loaded with miR-378a-3p accelerated osteolysis by targeting the Dyrk1a/Nfatc1 axis to upregulate ANGPTL2 expression and secretion, thereby promoting PMN formation and enhancing prostate cancer metastasis. Therefore, reducing the release of miR-378a-3p-containing EVs or inhibiting the recruitment of miR-378a-3p in EVs could be a therapeutic strategy to target prostate cancer metastasis [119]. It was found that blocking exosomal miR-3473b derived from lung cancer cells could reverse NF-κB activation of fibroblasts and reduce the formation of PMN and intrapulmonary colonization. So miR-3473b is a potential therapy target for metastasis of lung cancer [131]. Similarly, the blockage of exosomal miR-3157-3p derived from NSCLC cells maintained tight junctions between vascular endothelial and reduced PMN formation and subsequent NSCLC metastases, suggested that miR-3157-3p might be a therapeutic target to address NSCLC metastasis [61]. MiR-92a transported through EVs had the ability to promote HSC activation and expression of ECM proteins, which enhanced the accumulation of immunosuppressive cells and the attachment of tumor cells after remodeling the hepatic microenvironment, thereby providing a key mediator for PMN formation in the liver. In this way, miR-92a provided a valuable new target for interventional treatment of hepatic metastases of lung cancer [132]. Exosomal lncRNA-SOX2OT derived from NSCLC cell modulated osteoclast differentiation and stimulated bone metastasis, potentially becoming a new prognostic biomarker and therapeutic target for metastatic NSCLC [125].

In addition, EVs can also be used as drug delivery vehicles due to their natural, non-toxic, and biodegradable properties as well as their ability to cross various biological barriers, including the BBB. For example, the RNAi delivered by the bionic nano-drug delivery system (CBSA/siS100A4@exosome) from autologous breast cancer cells could effectively inhibit the upregulation of S100A4 protein associated with tumor metastasis in the PMN of lungs, thus effectively inhibited postoperative lung metastasis in early triple-negative breast cancer (TNBC) [133]. The above studies suggest that targeted blockade of associated EV-loaded ncRNA can be used as an interventional therapy for tumor metastasis, and EVs are gradually revealing their ability as drug carriers, which will make significant contributions to molecular-targeted therapy and precision medicine (Table 2).

## 5. Conclusions

NcRNAs carried by EVs are important members of intercellular communication, which promotes tumor PMN formation through different pathways [17,134]. The formation of PMN largely determines if circulating tumor cells can adhere, survive, proliferate, and eventually promote tumor-distant metastasis on the site to be metastasizing [135]. In recent years, there have been an increasing number of studies on ncRNAs carried by EVs associated with PMN. Even more remarkably though, EVs carry ncRNAs that are potentially valuable as a form of liquid biopsy in the diagnosis of tumor metastasis, and they may also provide targets for the treatment of tumor metastasis [136]. However, PMNs are in an extremely complex microenvironment, and there are still many unanswered questions that are still in the exploration stage. (1) What is the difference in the role of ncRNAs carried by tumor-derived EVs and ncRNAs carried by immune cells or stromal-cell-derived EVs in the PMN? (2) The composition of EVs is complex, so what role do these components play in PMN formation? Are there interactions between the different components? (3) Is the effect of ncRNA carried by EVs on PMN terminated after surgical resection of the primary tumor? (4) What are the reliability and validity of EV-carried ncRNAs as biomarkers of tumor-distant metastasis? (5) Some evidence suggests that the dormancy of tumor cells in PMN may be crucial in metastasis. EVs may mediate the reawakening of dormant niches, and the role of ncRNAs in this process is vital [78], which will provide a novel idea for the involvement of ncRNAs derived from EVs in distant tumor metastasis. Looking ahead, further understanding of the mechanism of PMN formation and its impact on tumor-distant metastasis will provide novel diagnostic targets and therapeutic strategies for the prevention and treatment of metastatic tumors.

## Figures and Tables

**Table 1:** NcRNAs carried by EVs may be biomarkers for predicting tumor metastasis.

Tumor	EVs Cargo Content	Utilization	Refs.
CRC	miR-17-5p, miR-92a-3p	provided a promising noninvasive prognostic biomarker for metastatic CRC	[113]
	miR-19a	identified as a prognostic biomarker	[114]
	miR-21	induced macrophage polarization toward an IL-6-secreting proinflammatory phenotype.	[42]
	miR-203	promoted the differentiation of monocytes to M2 TAMs	[46]
	miR-934	induced macrophage M2 polarization	[47]
	miR-25-3p	promoted angiogenesis and increased vascular permeability	[58]
	miR-181a-5p	activate HSCs	[75]
Breast cancer	miR-200	altered gene expression and promoted EMT	[117]
	miR-105	induced metastasis and vascular permeability in distant organs	[59]
	miR-218	regulated the collagen deposition by osteoblasts	[72]
	miR-200b-3p	promoted the inflammatory PMN formation	[43]
	let-7s	enabled neutrophil recruitment and N2 conversion	[50]
Prostate cancer	miR-378a-3p	promoted osteolysis	[119]
	miR-425-5p	biomarker for prostate cancer bone metastasis	[120]
GC	miR-23b	biomarker for detecting liver metastasis of GC	[121]
	miR-29b-3p circ-RanGAP1	plays pivotal role in tumor metastasis upregulated VEGFA expression.	[122] [123]
Lung cancer	miR-3157-3p	promoted angiogenesis and disrupted the tight junctions of venous endothelial cells	[61]
	miR-192 lncRNA-Sox2ot	increased angiogenesis and promoted osteolysis targeting regulated miR-194-5p	[124] [125]
HCC	miR-638	attenuated endothelial junction integrity	[60]
	miR-1247-3p	biomarker for HCC metastasis	[76]
NPC	miR-23a	regulated angiogenesis	[56]
Pancreatic cancer CCA	lncRNA-Sox2ot circ-CCAC1	competitively binds to the miR-200 family upregulated YY1	[126] [127]

Abbreviation: EVs, extracellular vesicles; miRNA, microRNA; CRC, colorectal cancer; IL, interleukin; TAMs, tumor-associated macrophages; HSCs, hepatic stellate cells; EMT, epithelial mesenchymal transition; PMN, pre-metastatic niche; GC, gastric cancer; VEGFA, vascular endothelial growth factor A; HCC, hepatocellular carcinoma; YY1, Yin Yang 1.

**Table 2:** Targeted intervention in tumor metastasis of ncRNAs carried by EVs.

Tumor	EVs Cargo Content	Utilization	Refs.
HCC GC CCA	circPTGR1 circRNA CDR1-AS circ-RanGAP1 circ-CCAC1	competitively with MET to target miR449a promoted the expression of AFP by sponging miR-1270 promoted GC progression by miR-877-3p/VEGFA axis reduced the levels of intercellular junction proteins	[128] [129] [123] [127]
CRC	miR-25-3P	promoted angiogenesis and increase vascular permeability	[58]
Breast cancer	miR-21	facilitated osteoclastogenesis through regulating PDCD4	[130]
Prostate cancer	miR-378a-3p	induced osteolytic progression	[119]
Lung cancer	miR-3473b	hindered the NFKBID function	[131]
	miR-3157-3p	destroyed tight junctions between vascular endothelial	[61]
	miR-92a lncRNA-Sox2ot	led to the enhancement of TGF-β signaling in HSCs promoted bone metastasis by miR-194-5p/RAC1 signalling axis	[132] [125]
Breast cancer	siRNA	CBSA/siS100A4 @ Exosome nanoparticles suppressed postoperative metastasis	[133]

Abbreviation: EVs, extracellular vesicles; HCC, hepatocellular carcinoma; MET, mesenchymal epithelial transition factor; AFP, alpha-fetoprotein; GC, gastric cancer; VEGFA, vascular endothelial growth factor A; CRC, colorectal cancer; miRNA, microRNA; PDCD4, programmed cell death 4; NFKBID, NF-κB inhibitor deltas; HSCs, hepatic stellate cells; RAC1, ras-related C3 botulinum toxin substrate 1.

## Data Availability

The data presented in this study are available in this article.
